# Dialyzer clearance of myoglobin with middle molecule filter and low blood flow CVVHD in patients with rhabdomyolysis-associated acute kidney injury

**DOI:** 10.1186/cc12370

**Published:** 2013-03-19

**Authors:** I Leppanen, T Ahonen, J Tenhunen

**Affiliations:** 1 Tampere University Hospital, Tampere, Finland; 2 Uppsala University Hospital, Uppsala, Sweden

## Introduction

Rhabdomyolysis-associated acute kidney injury can be treated with various modes of renal replacement therapy (RRT) [1,2]. We have used continuous venoveno hemodialysis (CVVHD) with middle molecule filter and low extracorporeal blood flow (100 to 180 ml/minute) to avoid albumin loss and hemodynamic instability.

## Methods

We treated nine patients suffering from rhabdomyolysis and acute kidney injury with various causes. We used a CVVHD mode with low (100 to 180 ml/minute) blood flow and a middle molecule filter (Ultraflux EMiC2; 1.8 m^2 ^surface area, polysulfone membrane, cutoff at 403kDa, blood filling volume 130 ml, blood flow range 100 to 350 ml/ minute, maximum dialysate flow 1,000 ml/minute). Myoglobin and albumin concentrations were measured from prefilter and postfilter samples and dialyzer clearances were calculated (CLdial = blood flow×Cpre - Cpost/Cpre + UF×Cpre/Cpost). Measurements were taken at 0, 15 minutes, 30 minutes, 4 hours, 12 hours, 24 hours, 36 hours and 48 hours from the start of the CRRT or until CRRT was no longer needed.

## Results

The baseline and prefilter plasma albumin concentrations varied from 8 to 29 g/l. Postfilter and post-treatment albumin concentrations remained comparable. Prefilter concentrations of myoglobin (17.8 kDa) varied from 96,109 to 747 μg/l and the dialyzer clearance of myoglobin from 50.6 to 0 ml/minute. The mean dialyzer clearance was 23.1 ml/minute and the median clearance 22.2 ml/minute. Maximal changes between corresponding prefilter and postfilter samples were: absolute concentration change 27,091 μg/l, percentage 34%, dialyzer clearance 50.6 ml/minute. Clearances were achieved with low extracorporeal blood flow between 100 and 180 ml/minute, most commonly 120 ml/ minute. Ultrafiltration (UF) was used only at five of the 56 time points calculated, because of hemodynamic instability. All patients required either vasopressor or vasopressor and inotrope support. The highest dialyzer clearance of myoglobin (50.6 ml/minute) was measured at the prefilter myoglobin of 29,266 μg/l, blood flow 180 ml/ml and no UF.

## Conclusion

In a rhabdomyolysis-associated kidney injury a middle molecule filter and a low blood flow CVVHD offer a safe and effective treatment choice for patients requiring vasopressor or vasopressor and inotrope for hemodynamic support.
